# Prevalence of Insomnia Symptoms in Older Chinese Adults During the COVID-19 Pandemic: A Meta-Analysis

**DOI:** 10.3389/fmed.2021.779914

**Published:** 2021-11-15

**Authors:** Qian-Qian Zhang, Lan Li, Bao-Liang Zhong

**Affiliations:** Affiliated Wuhan Mental Health Center, Tongji Medical College of Huazhong University of Science and Technology, Wuhan, China

**Keywords:** insomnia, older adults, prevalence, COVID-19, China

## Abstract

**Background:** The ongoing COVID-19 pandemic has disproportionately affected the sleep health of older adults, but the limited number of studies on insomnia symptoms of older Chinese adults differed in terms of screener of insomnia, sample size, and prevalence, making mental health planning for this population difficult. This meta-analysis estimated the prevalence of insomnia symptoms in older Chinese adults during the COVID-19 pandemic.

**Methods:** Both Chinese (CNKI, Wanfang, VIP) and English (PubMed, EmBase, PsycInfo) databases were systematically searched to identify cross-sectional studies containing data on the prevalence of insomnia symptoms in older Chinese adults during the pandemic. Risk of bias (RoB) of included studies was assessed with the Joanna Briggs Institute Critical Appraisal Checklist for Studies Reporting Prevalence Data.

**Results:** Nine studies with a total of 27,207 older Chinese adults were included. RoB scores of these studies ranged between zero and six. The pooled prevalence rates of insomnia symptoms and moderate and severe insomnia symptoms were 24.6% [95% confidence interval (CI): 19.5–30.5%] and 11.1% (95% CI: 7.2–16.9%), respectively. In subgroup analysis, significantly higher prevalence rates were observed in studies defining insomnia symptoms as “Insomnia Severity Index (ISI) ≥ 8” than in those defining them as “ISI ≥ 15” (32.6 vs. 15.6%, *P* < 0.001) and in older adults living in the COVID-19 epicenter than in those living in other places (35.2 vs. 23.3%, *P* = 0.006).

**Conclusion:** Nearly one out of every four older Chinese adults suffered from insomnia symptoms during the pandemic. Mental health services for this population during the pandemic should include supportive activities aimed at improving mental well-being, periodic assessment of insomnia symptoms, and psychiatric assessment and treatment when necessary.

## Introduction

Sleep disturbances and insomnia are common among older adults. In China, 35.9% of the adults aged 60+ years suffer from sleep disturbances and 24.4–26.8% report insomnia symptoms in the most recent month, as defined by a cut-off score on the Pittsburgh Sleep Quality Index (PSQI) (1–3). Factors associated with late-life insomnia symptoms included no spouses, mental health problems, major medical conditions, negative life events, and acute or recurring psychosocial stressors (2–5).

The ongoing COVID-19 pandemic is a significant source of stress in people's lives and has profound negative effects on the mental health of people of all ages (6). Because the majority of COVID-19-related hospitalizations and deaths occur in persons aged 65+ years, older adults have been disproportionately affected by the pandemic (7). The current public health guidelines recommend older adults to keep social distancing with others and stay indoors as much as possible, which in turn result in elevated levels of loneliness and social isolation in older adults (8, 9). Importantly, in the context of the pandemic, older adults are confronted with disruptions to the daily routines due to lockdown, difficulties in timely accessing to needed healthcare services due to the overwhelmed hospitals, financial loss due to economic recession, separation from family members due to mass quarantine, and difficulties in using digital technologies due to lack of internet access and no smartphones (10–12); these associated stressors may further exacerbate the mental health of older adults.

Mental health problems, including insomnia, are a common stress reaction following exposure to a traumatic event such as the COVID-19 pandemic. Nevertheless, compared to the numerous studies on mental health and sleep problems in general adults during the pandemic (13–15), relatively fewer have investigated sleep problems in the elderly population.

China has the world's largest population of older adults and Chinese adults aged 65+ years will rise to 366 million in 2050, accounting for 26.1% of the total population (16). Since the reform and open door policy in 1978, substantial socioeconomic–cultural changes have challenged the mental health of older adults in China. For example, the massive rural-to-urban migration of young labors has resulted in millions of left-behind older adults, the one-child policy and the erosion of the traditional family structure have resulted in millions of empty-nest elders, and the diminishing traditional value of filial piety has reduced adult children's willingness to care for older adults (17, 18). Therefore, the ongoing pandemic coupled with the rapid aging may have a much more devastating toll on the mental and sleep health of older Chinese adults.

In China, a few population-based studies have examined the prevalence of insomnia symptoms in older adults amid the pandemic, but these studies varied in terms of screener of insomnia [i.e., PSQI vs. Insomnia Severity Index (ISI)], sample size (i.e., from 35 to 13,964), and prevalence (i.e., from 13.1 to 42.9%) (19–23). Importantly, nearly all available data on older adults are derived from whole population-based studies, where elderly-specific prevalence data are not easily accessed (i.e., only shown in the main text). To facilitate the development of mental health policy aimed at preventing or reducing insomnia in older Chinese adults during the pandemic, it is necessary to systematically review available studies and estimate the prevalence of insomnia symptoms.

## Materials and Methods

This systematic review and meta-analysis was reported according to the Preferred Reporting Items for Systematic Reviews and Meta-Analyses (PRISMA) guideline (Supplementary Table 1). Two authors (QQ Zhang and L Li) independently searched literature, extracted data, and performed risk of bias (RoB) assessment. Any disagreements were resolved by a discussion with the correspondence author (BL Zhong). In this study, after cross-checking, no discrepancies were found in the results of literature search, study selection, and data extraction. However, the two authors had different RoB scores on two included studies. After discussion with the correspondence author, consensus was achieved across the three authors.

### Literature Search

Potential studies published between January 1, 2020 and September 17, 2021 were searched in both Chinese and English databases: Chinese National Knowledge Infrastructure (CNKI), Wanfang data, VIP Information, PubMed, EmBase, and PsycInfo. The search terms were as follows: (sleep disturbance OR sleep quality OR insomnia OR sleep problem OR sleep disorder OR sleep symptom) AND (epidemiology OR cross-sectional study OR prevalence OR rate) AND (coronavirus disease 2019 OR 2019-n-CoV OR severe acute respiratory syndrome coronavirus 2 OR COVID-19 OR COVID) AND (China OR Chinese). To avoid missing studies that focused on the general population but simultaneously presented elderly-specific data in their main texts, we had no restrictions on participants in the search strategy. Further manual search was performed among the references of selected papers and related reviews.

### Study Selection

Studies were included if they satisfied the following criteria: (a) participants were general older Chinese adults aged 50 years or above; (b) reported the prevalence of insomnia symptoms, as measured by validated screeners of insomnia, including PSQI, ISI, and Athens Insomnia Scale (AIS); and (c) cross-sectional or cohort studies (only the baseline data were extracted) that were conducted in China during the COVID-19 pandemic. We also included general population-based studies that separately presented elderly-specific prevalence of insomnia symptoms. We excluded studies focusing on special populations such as COVID-19 patients, older adults living in long-term care facilities, and old patients with major medical conditions, as well as those using unstandardized assessments of insomnia. Currently, there is no agreement on the cut-off age to define older adults (24). Following several previous studies (25, 26), older adults were defined as those aged 50 years or older in the present study. Because PSQI has been widely used as a screener of insomnia and it has comparable accuracy for insomnia screening compared to ISI and AIS (27, 28), PSQI is suitable for detecting the presence of insomnia symptoms.

### Data Extraction

We extracted the following information from included studies: first author, publication year, study site, survey date, survey method, sampling approach, sample size, insomnia screener, and rate of insomnia symptoms.

### RoB Assessment of Included Studies

The Joanna Briggs Institute Critical Appraisal Checklist for Studies Reporting Prevalence Data (“JBI checklist” hereafter) was used to measure the level of RoB of included studies (29). The JBI checklist has nine methodology domains: sample frame, sampling, sample size, description of participants and setting, sample coverage of the data analysis, validity of the instrument for assessing the outcome, standardization and reliability of the instrument for assessing outcome, statistical analysis, and response rate (30). Each domain has four options (yes, no, unclear, or not applicable) and one point is assigned to a “yes” answer. The total RoB score ranges between zero and nine, with a higher score denoting a lower RoB.

### Statistical Analysis

We used the “metaprop” package of R, version 4.0.2, to conduct the meta-analysis. *I*^2^ statistic was used to test heterogeneity between studies and, when there was evidence of heterogeneity (*I*^2^ > 50%), a random-effect model was used to generate the pooled estimate of prevalence. Subgroup analysis was used to explore the source of heterogeneity in the prevalence estimate of insomnia symptoms. Publication bias was assessed by funnel plots, Egger's regression model, and Begg's test. Because Generalized Linear Mixed Models (GLMMs) perform better than the conventional two-step approach when small-sample studies are included for synthesizing proportions, GLMMs were used to directly model event counts without data transformation within studies (31). Sensitivity analysis was conducted by removing each study individually to assess the robustness of pooled estimate of prevalence. *P* < 0.05 (two-sided) was deemed as statistically significant.

## Results

**Figure 1:**
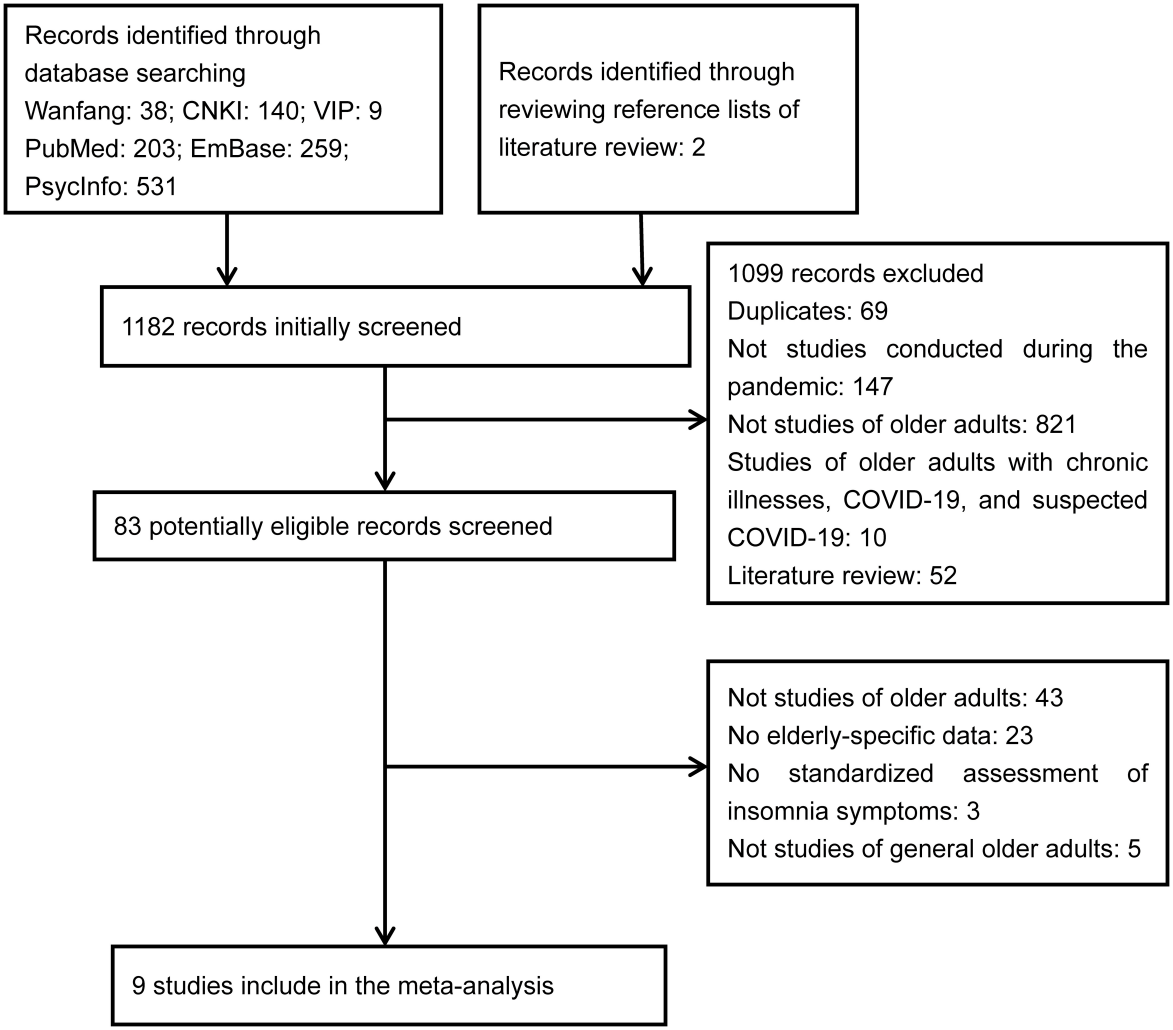
Flowchart of study inclusion.

Altogether, nine studies with 27,207 older Chinese adults were eligible and included (Figure 1) (19–23, 32–35). All studies assessed the presence of insomnia symptoms in convenient samples of older adults during the outbreak period of COVID-19 in China. With regard to the insomnia screener, five studies used ISI, three used PSQI, and one used AIS. Detailed characteristics of included studies are displayed in Table 1.

**Table 1:** Characteristics of studies included in the meta-analysis.

**References**	**Site**	**Survey date**	**Sampling method**	**Sample size**	**Males, *n* (%)**	**Age of older adults, years**	**Survey method**	**Assessment of insomnia**	**No. of old adults with insomnia symptoms (prevalence, %)**	**Score of risk of bias**
Gao et al. (34)	Ya'an, China	February 10–15, 2020	Convenience sampling	37	Not reported	≥55	Online self-administered questionnaire	PSQI > 7	9 (24.3)	3
Liu et al. (20)	Mainland China	February 1–10, 2020	Convenience sampling	231	120 (51.9)	≥51	Online self-administered questionnaire	PSQI > 7	53 (22.9)	5
Wang et al. (32)	Mainland China	February 2–18, 2020	Convenience sampling	1,209	Not reported	≥50	Online self-administered questionnaire	ISI ≥ 8	432 (35.7)	4
Wang et al. (33)	Mainland China	February 4–18, 2020	Convenience sampling	781	Not reported	≥50	Online self-administered questionnaire	PSQI > 7	190 (24.3)	5
Wang et al. (23)	Mainland China	February 10–17, 2020	Convenience sampling	7,177	Not reported	≥50	Online self-administered questionnaire	ISI ≥ 15	940 (13.1)	5
Wang et al. (22)	Mainland China	March 16–29, 2020	Convenience sampling	13,964	Not reported	≥50	Online self-administered questionnaire	ISI ≥ 15	2,576 (18.4)	5
Zhang et al. (21)	Wuhan, China	February 1–5, 2020	Convenience sampling	35	Not reported	≥51	Online self-administered questionnaire	ISI ≥ 8	15 (42.9)	3
Zheng et al. (19)	Mainland China	February 28–March 11, 2020	Convenience sampling	3,730	1,704 (45.7)	≥50	Online self-administered questionnaire	ISI ≥ 8	1,027 (27.5)	6
Zhou et al. (35)	Mainland China	February 23–March 1, 2020	Convenience sampling	43	Not reported	≥60	Online self-administered questionnaire	AIS ≥ 6	12 (27.9)	3

*ISI, Insomnia Severity Index; PSQI, Pittsburgh sleep Quality Index; AIS, Athens Insomnia Scale*.

The JBI checklist scores of the studies ranged between three and six. No studies were rated as low RoB (JBI checklist score = 9) (Table 1). The three most common limitations were inappropriate way of sampling of participants (9/9), unclear response rates of study samples (9/9), and unclear information for assessing whether the outcome was measured in a standard, reliable way for all participants (9/9).

**Figure 2:**
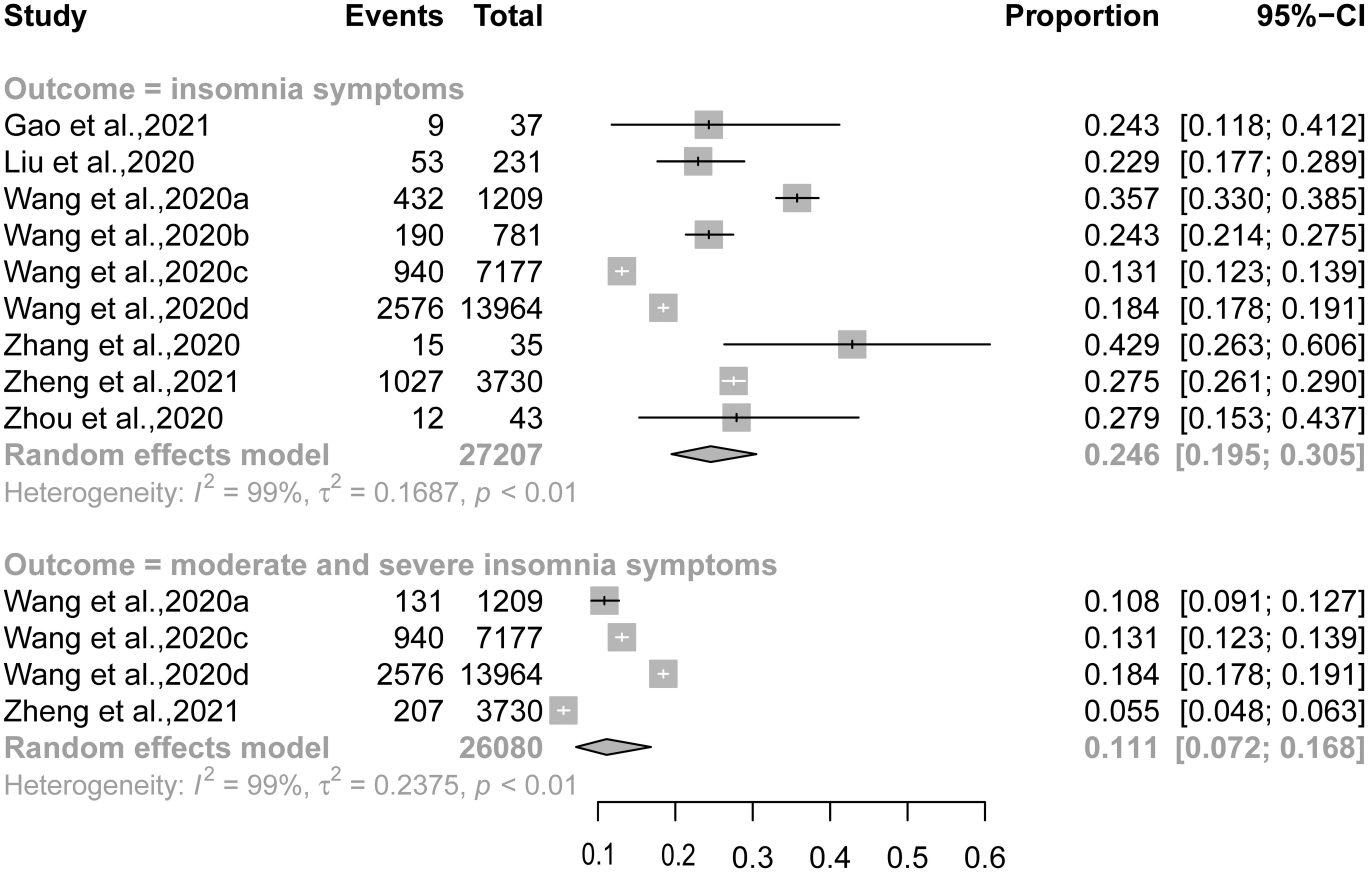
Forest plot of the prevalence of insomnia symptoms and moderate and severe insomnia symptoms.

The pooled prevalence rates of insomnia symptoms and moderate and severe insomnia symptoms were 24.6% [95% confidence interval (CI): 19.5–30.5%] and 11.1% (95% CI: 7.2–16.9%), respectively (Figure 2). Results of subgroup analysis showed that significantly higher prevalence rates were observed in studies defining the presence of insomnia symptoms as “ISI ≥ 8” than in those defining it as “ISI ≥ 15” (32.6 vs. 15.6%, *P* < 0.001) and in studies carried out in the COVID-19 epicenter than in those in other places (35.2 vs. 23.3%, *P* = 0.006) (Table 2).

**Table 2:** Subgroup analysis of the source of heterogeneity of included studies.

**Characteristics**		**No. of studies**	**Sample size**	**No. of older adults with insomnia symptoms**	**Heterogeneity, *I*^**2**^ (%) (*P*)**	**Pooled prevalence (95%CI), %**	**Q**	***P***
Assessment	ISI	5	26,115	4,990	99.3 (<0.001)	25.1 (17.0, 35.4)	Rreference	
	PSQI	3	1,049	252	0.00	24.0 (21.5, 26.7)	0.050	0.824
	ASI	1	43	12	Not applicable	27.9 (16.6, 43.0)	0.120	0.733
Cut-off score of ISI	≥8	3	4,974	1,474	93.8 (<0.001)	32.6 (26.8, 39.0)	Rreference	
	≥15	2	21,141	3,516	99.0 (<0.001)	15.6 (12.3, 19.7)	22.640	<0.001
Site	Hubei, China	2	182	64	10.4 (<0.001)	35.2 (28.6, 42.4)	Rreference	
	Others	8	27,025	5,190	98.7 (<0.001)	23.3 (18.5, 28.7)	7.470	0.006
Age, years	≥50	7	27,127	5,233	98.9 (<0.001)	24.5 (18.6, 31.4)	Rreference	
	≥55	2	80	21	0.00	26.3 (17.8, 36.9)	0.090	0.760
Risk of bias score	0–3	3	115	36	37.1 (<0.001)	31.3 (23.4, 40.4)	Rreference	
	4–6	6	27,092	5,218	99.1 (<0.001)	22.9 (17.5, 29.2)	2.650	0.104
Sample size	<506	4	346	89	51.5 (<0.001)	27.0 (20.1, 35.2)	Rreference	
	≥506	5	26,861	5,165	99.3 (<0.001)	22.9 (16.7, 30.5)	0.620	0.431

*ISI, Insomnia Severity Index; PSQI, Pittsburgh sleep Quality Index; AIS, Athens Insomnia Scale*.

**Figure 3:**
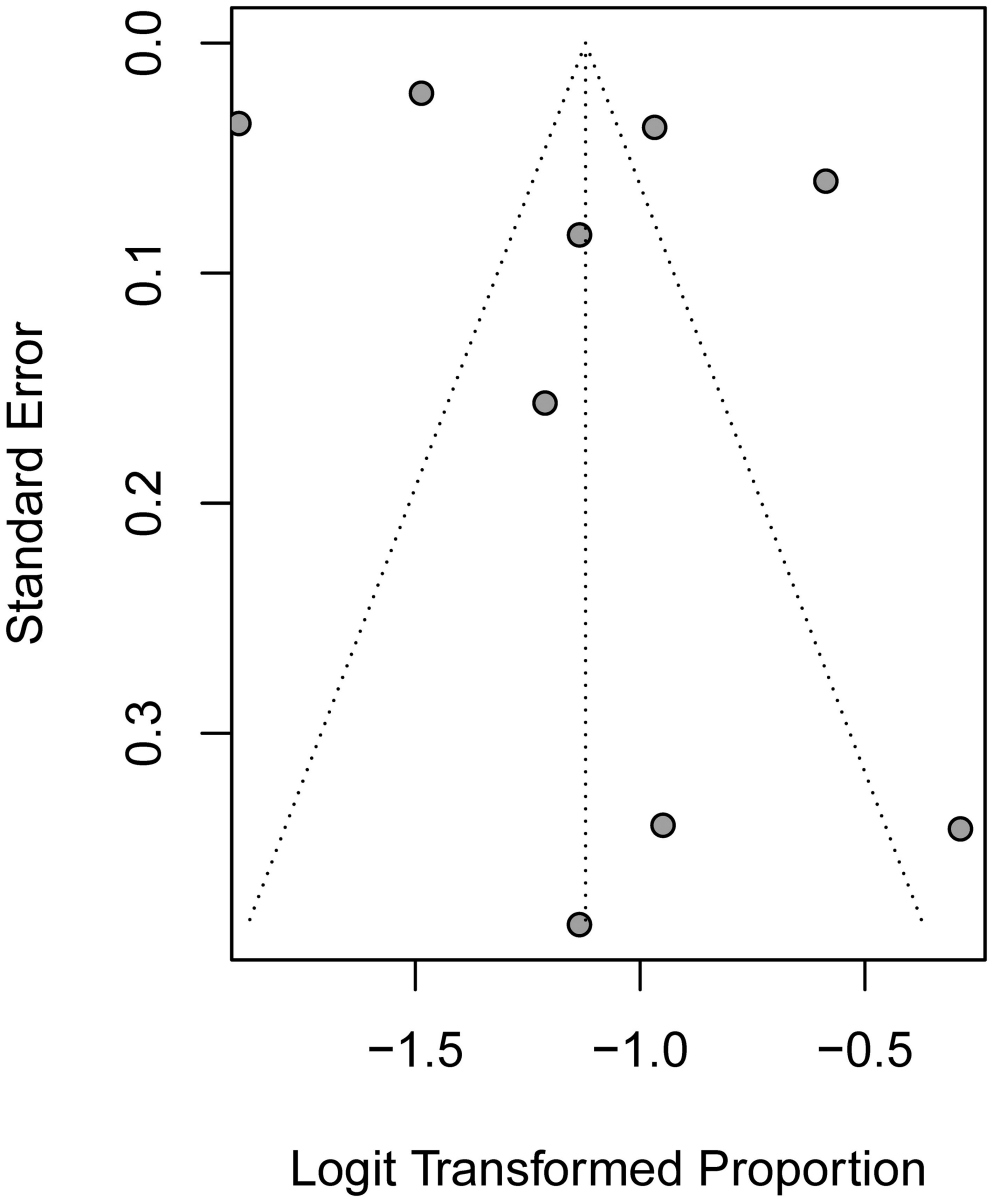
Funnel plot of publication bias among the nine included studies.

The Egger's test (*t* = 0.97, *P* = 0.365) and Begg's test (*z* = 0.210, *P* = 0.835) revealed no statistically significant publication bias (Figure 3).

**Figure 4:**
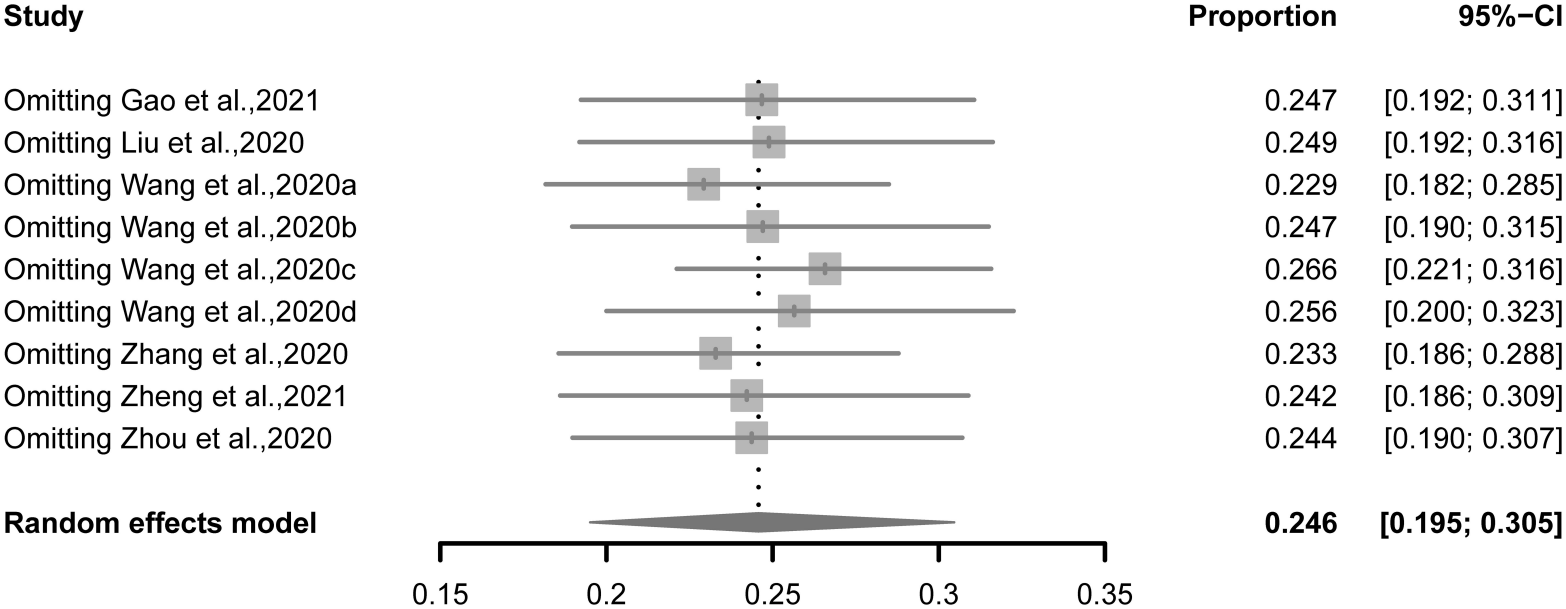
Sensitivity analysis of the pooled estimate of prevalence in this meta-analysis.

When each study was excluded sequentially, the pooled estimate of prevalence (22.9–26.6%) and heterogeneity across studies (*I*^2^: 97.7–98.8%) were not altered significantly, suggesting that no outlying study could influence the overall results of the meta-analysis (Figure 4).

## Discussion

This meta-analysis quantitatively summarized studies estimating the prevalence of insomnia symptoms in older adults in China amid the COVID-19 pandemic. We found an overall prevalence rate of 24.6% of insomnia symptoms in older Chinese adults and significantly higher rates in studies defining insomnia symptoms as “ISI ≥ 8” (vs. “ISI ≥ 15”) and older adults living within the COVID-19 epicenter (vs. other places). In addition, 11.1% of the older Chinese adults had moderate and severe insomnia symptoms.

Compared to the 24.4–26.8% prevalence of insomnia symptoms among older Chinese adults during the non-pandemic era (1–3), a comparable prevalence of insomnia symptoms (24.6%) was found in older Chinese adults during the pandemic. This estimate seems to not support the additional risk of insomnia exerted by the pandemic. Nevertheless, we argue that the result from this direct comparison should be considered cautiously owing to the significant heterogeneity in the methodologies of included studies. For example, most studies included in this meta-analysis defined older adults as those aged 50 years or over but the aforementioned 24.4–26.8% prevalence estimate was derived from studies of older adults aged 60 years or above (1–3). Because there is evidence showing an increasing prevalence of insomnia symptoms with increasing age in older Chinese adults (3, 36), our study should have a higher prevalence estimate of insomnia symptoms in older adults during the pandemic if older adults were those aged 60+ years. Moreover, as displayed in Table 2, when studies were limited to those defining the presence of insomnia symptoms as “ISI ≥ 8,” the synthesized prevalence rose to 32.6%, which is higher than the aforementioned 24.4–26.8% prevalence in older adults during the non-pandemic era (1–3). In addition, in our study the 11.1% prevalence of moderate and severe insomnia symptoms indicates that nearly a half (11.1/24.6%) of the older adults with insomnia symptoms are severe enough for clinical attention. These data may suggest an elevated risk of insomnia symptoms in older Chinese adults during the COVID-19 pandemic.

In the literature, living in Hubei or its capital city, Wuhan, was significantly associated with insomnia symptoms in both the general population and older adults in China (19, 37). In accordance with these studies, our meta-analysis confirmed the significantly higher risk of insomnia symptoms in older adults living within the COVID-19 epicenter than in those living in other places. This may be ascribed to the longer duration of mass quarantine, more strict social distancing measures, and higher risk of exposure to COVID-19 in persons living within than those living outside the epicenter (12).

The strength of this meta-analysis is the large sample size of older Chinese adults, which provided an overall and reliable estimate of the prevalence of insomnia symptoms in this vulnerable population during the COVID-19 pandemic. Nevertheless, this study also has a few limitations. First, none of the included studies were of low RoB. Because subgroup analysis according to RoB score revealed a trend toward lower prevalence of insomnia symptoms in studies with higher RoB scores (Table 2), we may have overestimated the true prevalence of insomnia symptoms in older Chinese adults during the pandemic. However, as we argued above, several included studies used strict criteria to define the presence of insomnia symptoms (i.e., “ISI ≥ 15”), so we may have underestimated the true prevalence of insomnia symptoms. Given the two limitations, it is difficult to evaluate the extent and direction of bias in the prevalence estimate. Second, since sex-specific and region-specific (i.e., urban vs. rural) prevalence data were available in only one of the included studies (19), this meta-analysis was not able to provide data on the demographic characteristics of insomnia symptoms in older adults. Third, all of the included studies were conducted during the COVID-19 outbreak period, so it remains unclear whether the sleep quality of older adults improves or worsens during the post-outbreak period. Finally, data on healthcare services utilization of older adults with moderate and severe insomnia symptoms are important for mental health policy-making during the pandemic, but the included studies had no such data.

In summary, nearly one out of every four older Chinese adults suffered from insomnia symptoms, suggesting a high level of mental healthcare need in this population in the context of COVID-19 pandemic. Insomnia has been associated with a range of physical and mental morbidities and elevated mortality in older adults (4). Given the high prevalence of insomnia symptoms, mental health services for this population during the pandemic should include supportive activities aimed at improving mental well-being, periodic assessment of insomnia symptoms to ensure early recognition of older adults who meet the diagnostic criteria for clinical insomnia, and psychiatric assessment and treatment when necessary. The higher prevalence rate of insomnia symptoms in older adults living in the epicenter indicates that more mental health resources should be assigned to older adults in the COVID-19 epicenter. In addition, prospective studies are warranted to understand the longitudinal changes of insomnia symptoms in older Chinese adults during the post-outbreak era.

## Data Availability Statement

The datasets presented in this study can be found in online repositories. The names of the repository/repositories and accession number(s) can be found in the article/Supplementary Material.

## Author Contributions

Q-QZ: acquisition and analysis of data for the study, drafting the paper, and interpretation of data for the study. LL: design and acquisition of data for the study. B-LZ: drafting the paper, revising the paper for important intellectual content, and interpretation of data for the study. All authors contributed to the article and approved the submitted version.

## Funding

This work was supported by National Natural Science Foundation of China (Grant Number: 71774060) and 2015 Irma and Paul Milstein Program for Senior Health Awards from the Milstein Medical Asian American Partnership Foundation. The two funding sources listed had no role in study design; in the collection, analysis and interpretation of data; in the writing of the report; and in the decision to submit the paper for publication.

## Conflict of Interest

The authors declare that the research was conducted in the absence of any commercial or financial relationships that could be construed as a potential conflict of interest.

## Publisher's Note

All claims expressed in this article are solely those of the authors and do not necessarily represent those of their affiliated organizations, or those of the publisher, the editors and the reviewers. Any product that may be evaluated in this article, or claim that may be made by its manufacturer, is not guaranteed or endorsed by the publisher.
